# Biomedical applications of dendritic fibrous nanosilica (DFNS): recent progress and challenges

**DOI:** 10.1039/d0ra04388e

**Published:** 2020-10-08

**Authors:** Mina Shaban, Mohammad Hasanzadeh

**Affiliations:** Pharmaceutical Analysis Research Center, Tabriz University of Medical Sciences Tabriz Iran hasanzadehm@tbzmed.ac.ir; Food and Drug Safety Research Center, Tabriz University of Medical Sciences Tabriz Iran

## Abstract

Dendritic fibrous nanosilica (DFNS), with multi-component and hierarchically complex structures, has recently been receiving significant attention in various fields of nano-biomedicine. DFNS is an emerging class of mesoporous nanoparticles that has attracted great interest due to unique structures such as open three-dimensional dendritic superstructures with large pore channels and highly accessible internal surface areas. This overview aims to study the application of DFNS towards biomedical investigations. This review is divided into four main sections. Sections 1–3 are related to the synthesis and characterization of DFNS. The biomedical potential of DFNS, such as cell therapy, gene therapy, immune therapy, drug delivery, imaging, photothermal therapy, bioanalysis, biocatalysis, and tissue engineering, is discussed based on advantages and limitations. Finally, the perspectives and challenges in terms of controlled synthesis and potential nano-biomedical applications towards future studies are discussed.

## Abstract 2

Dendritic fibrous nanosilica (DFNS) , with multi-component and hierarchically complex structures, has recently been receiving significant attention in various fields of nano-biomedicine.

## Graphical abstract

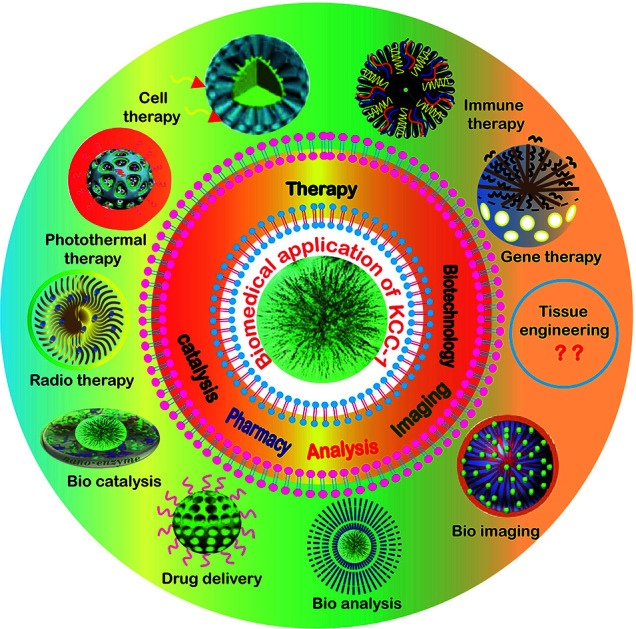

## 1. Introduction

Scientists have been continuously trying to find new ways to serve the needs of medical science. Nanotechnology provides the ability to manipulate the structure of materials on the atomic and molecular scale to produce new materials with new features that can afford solutions to some medical challenges.^1^ Recently, the development of structures of mesoporous nanomaterials has attracted attention in biomedical fields such as diagnostics, treatment, drug delivery and bioanalysis.^2–4^ Numerous nano-biomedical studies have been conducted on nanocomposites such as liposomes, polymers, nanoparticles, dendrimers, nano-micelles, nanogels, exosomes, magnetic nanoparticles (MNPs), mesoporous silica nanoparticles (MSNs), carbon nanotubes, and gold nanoparticles.^5–7^ Among these, a new generation of mesoporous nanomaterials, such as dendritic fibrous silica nanoparticles (DFSN), has received biomedical attention due to their special structural properties and intrinsic silica features. These features include easy synthesis, adjustable and uniform pore sizes, tunable particle sizes, unique large surface areas, great permeability, high-level pore volumes, low density, high thermal stability, great mechanical stability, highly selective functionalization of the pore surface or particle surface, low range of toxicity, and perfect biocompatibility.^8,9^ There are several types of mesoporous nanoparticles, including dendritic fibrous nanosilica (DFNS) particles, MCM-41 (Mobil Composite Material), SBA-15 (Santa Barbara Amorphous), Stöber silica, mesosphere silica nanoparticles (MSNP), all of which have some advantages and disadvantages in nanomedicine application. Table 1 compares the physicochemical properties and cytotoxicities of different types of mesoporous nanoparticles.^10,11^ DFNS are fibrous spheres with higher temperature stability and smaller pore size, pore volume and size as compared to other mesoporous nanoparticles; cytotoxicity studies have shown that they have low toxicity.^11–13^ These properties allow them to be suitable in various nanomedical fields such as therapy, pharmacy, bioanalysis, imaging, biocatalysis and biotechnology. In recent years, several types of DFNS have been introduced for nano-biomedical applications due to their special structures. These include dendritic mesoporous silica nanoparticles (DMSNs),^14–17^ fibrous mesoporous silica microspheres(FMSMs),^18^ wrinkled silica nanoparticles (WSNs),^19,20^ wrinkled silica nanospheres (WSNs),^20^ wrinkled mesoporous silica (WMS),^21–23^ wrinkled mesoporous silica nanoparticles (WMSNs),^21,24^ radial-like mesoporous silica (RMS),^25,26^ fibrous silicon dioxide spheres (FSSs),^27^ fibrous silica nanoparticles or nanospheres (FSNs),^28,29^ dendrimer-like mesoporous silica nanoparticles with hierarchical pores (HPSNs),^30^ hierarchical and radial mesoporous (HRM),^31,32^ hierarchically structured spherical mesoporous nanofibrous (HSMNFs),^33^ wrinkle-structured periodic mesoporous organosilica (PMO),^34,35^ and mesostructured silica nanoparticles (MSNs).^36^ The history of DFNS exploration indicates that novel DFNS or KCC-1 was introduced by Polshettiwar *et al.* at KAUST (King Abdullah University of Science and Technology) Catalysis Center. They named it KCC-1 according to the acronym of their institution.^37^ Compared to other mesoporous silica nanocarriers, dendritic fibrous silica nanoparticles (DFSNs) with pore size ranging from 2 to 30 nm are perfect candidates for drug delivery and biomedical applications.^36^ The fibrous surface of DFNS allows them to be easily loaded with several ranges of organic groups, organometallic complex ionic liquids, polymers, peptides, enzymes, DNA, genes, drugs, *etc*.^10^ They can also interact with metal nanoparticles, bimetallic nanoparticles, as well as with single atoms of metals, quantum dots, metal oxides and hydroxides. These unique properties of DFNS allow them to be useful in a variety of applications such as catalysis,^38,39^ biomedical fields,^40^ analysis,^41^ industrial fields^42^ as adsorbents for water and air treatment,^43,44^ energy storage,^10^*etc*. Therefore, in this review, we have probed the spherical shape of DFNS with tunable particle sizes ranging from 14 to 1100 nm, their fibrous structure, which provides better accessibility to the DFNS surface, and their open structure and accessibility, which also permit the high loading of active molecules that make them suitable for use in biomedical fields. The toxicity studies of DFNS were conducted in animals but clinical trials have not been done to date.^45^ We also introduce DFNS as new nanocomposites with expanded biomedical applications. There are four main sections. Sections 1–3 are related to the synthesis and characterization of DFNS. In Section 4, the biomedical potential of DFNS, such as cell therapy, gene therapy, immune therapy, drug delivery, imaging, photothermal therapy, bioanalysis, biocatalysts, and tissue engineering, is discussed based on the advantages and limitations. Finally, perspectives and challenges in terms of the controlled synthesis and potential nano-biomedical applications towards future studies are discussed.

**Table 1:** A comparison of the physicochemical properties and cytotoxicities of DFNS, MCM-41, SBA-15, Stöber silica and MSN

Name	DFNS	MCM-41	SBA-15	Stöber silica	MSN
Pore structure	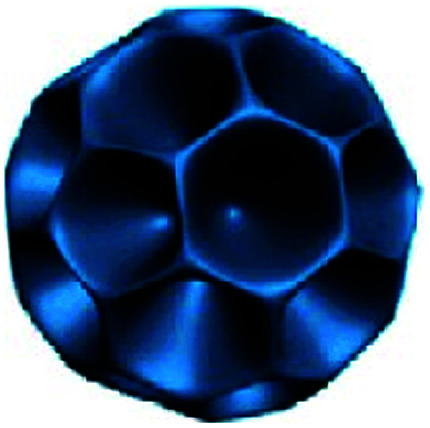	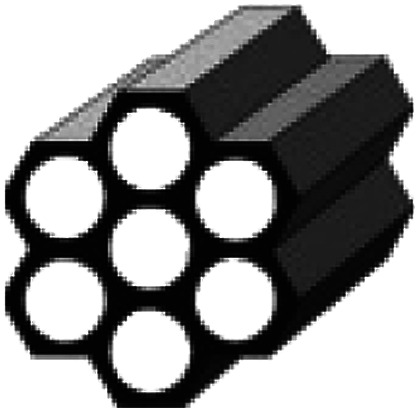	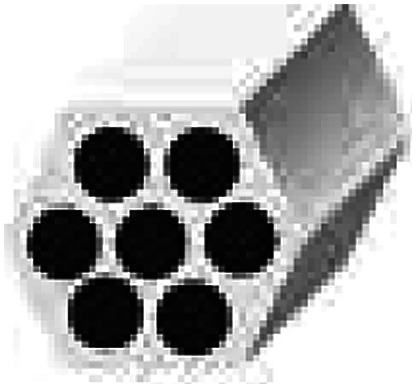	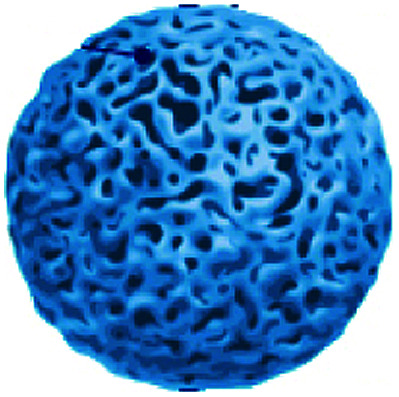	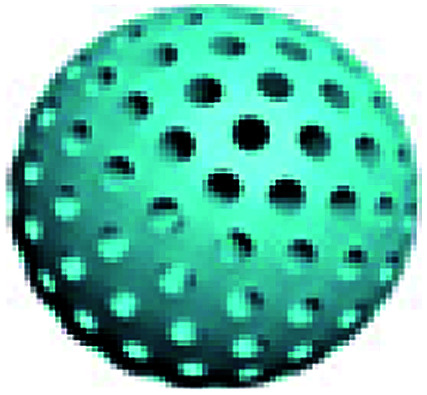
TEM	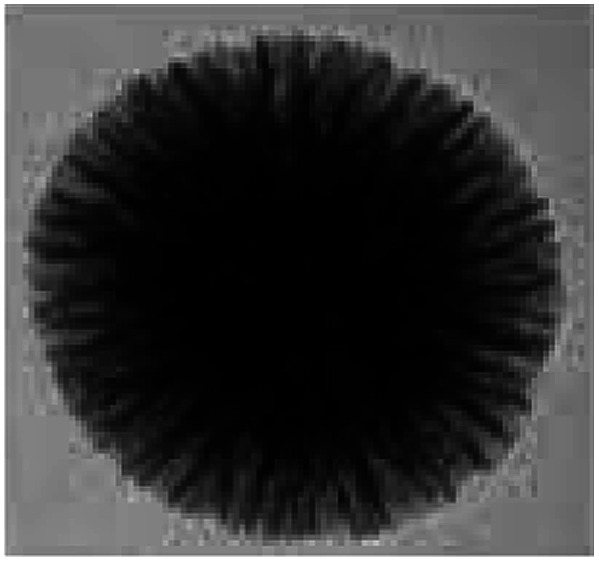	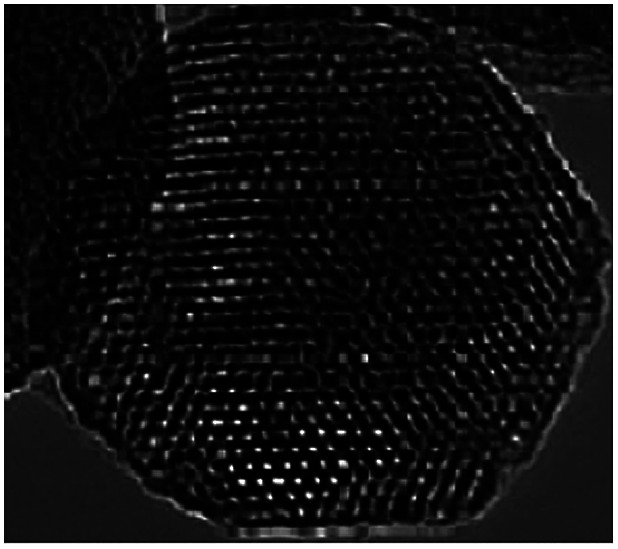	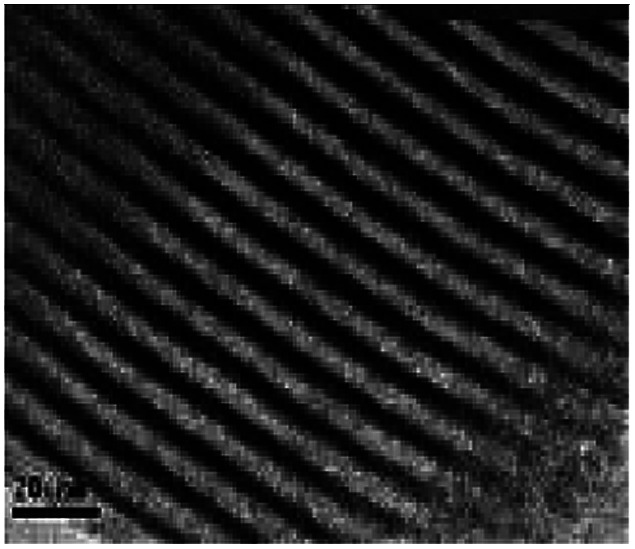	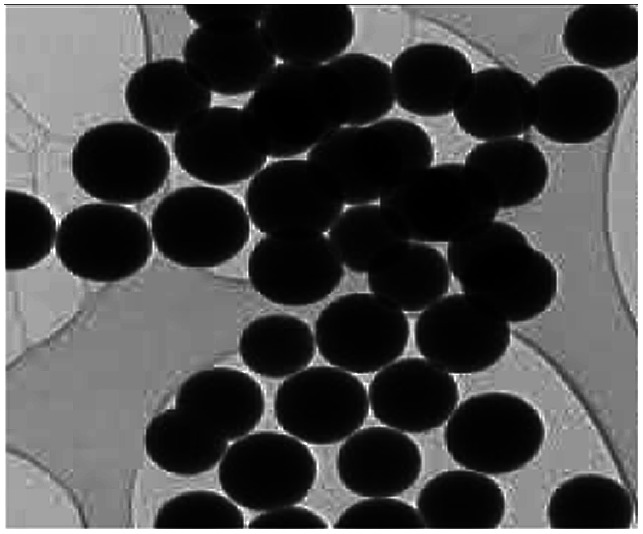	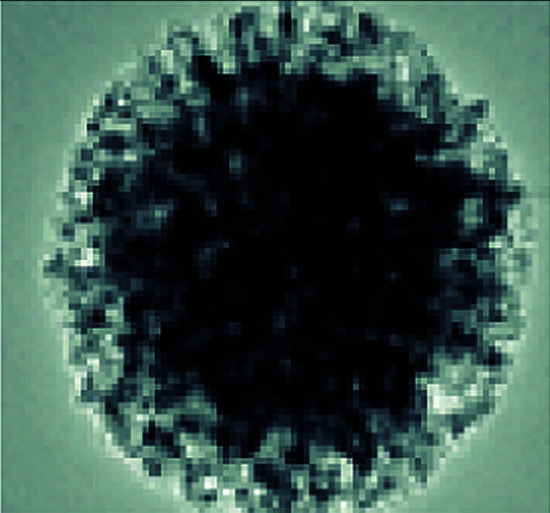
Pore sizes (nm)	3–25	30	5–50	1.2–5.9	2.88–9.92
Pore volume (cm^3^ g^−1^)	0.54–2.18	0.7–1.2	0.75–1.15	0.017–0.217	0.85–0.95
Thermal stability (°C)	950	707	600	NA	NA
Particle size (nm)	14–1110	Bulk (μm)	Bulk (μm)	20–3000	78–443
Cytotoxic potential	Low	Low	Low	High	Low

## 2. Synthesis of DFNS

### 2.1. Hydrothermal synthesis

**Fig. 1:**
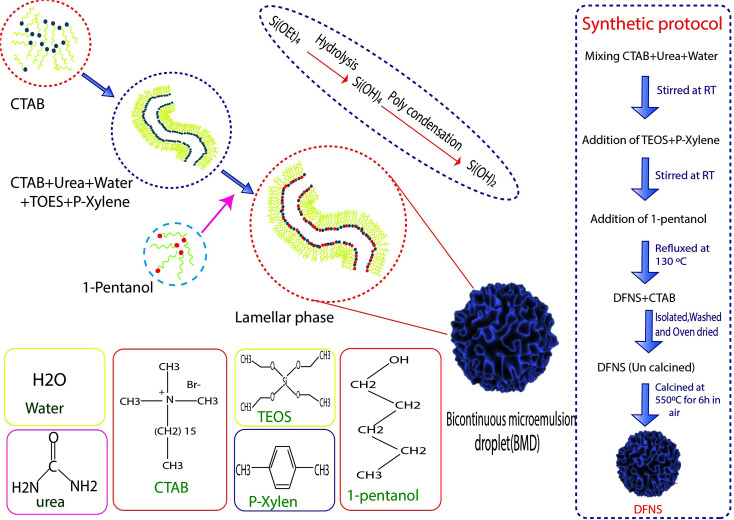
Graphic illustration of DFNS synthesis.

Hydrothermal synthesis involves the essential techniques of crystallizing substances from high-temperature aqueous solutions at high vapor pressures, mostly used for the fabrication of silicone monomers on the surface of NPs. On the other hand, this method is used for growing crystals of many different materials such as quartz, malachite, *etc.* This technique has also been used to grow dislocation-free single crystal particles, and the grains formed in this process could have better crystallinity than those from other processes.^46^ The process was based on minerals dissolving at high temperatures and pressures in water and subsequently crystallizing from dissolved materials.^41^ Polshettiwar *et al.* succeeded in synthesizing DFNS in their laboratory (KAUST Catalysis Center (KCC-1)), which were named KCC-1. Fig. 1 shows the stages of the synthesis of DFNS. KCC-1 was prepared using the microwave-assisted hydrothermal technique in a short time. Firstly, 0.012 mol solution of tetraethyl orthosilicate (TEOS) was dissolved in 30 ml solution of cyclohexane and 1.5 ml of pentanol. Then, another solution containing 0.0026 mol of cetyl pyridinium bromide (CPB) and 0.01 mol of urea in 30 ml of water was added to the first solution. This mixture was stirred for 30 min at 25 °C, and was placed in a teflon-sealed microwave (MW) reactor. The reaction mixture was exposed to MW irradiation (400 W maximum power) at 120 °C for 240 min. When the reaction was complete, the mixture was allowed to cool at room temperature and the silica formed was separated by centrifugation, then washed with distilled water and acetone, and air-dried for 24 h. The obtained material was calcined at 550 °C for 360 min in air.^37^ Another synthesis protocol was introduced in 2019 by Polshettiwar *et al.*, who showed that the previous protocol had some limitations. According to them, increasing the size above 800 nm *via* the previous method is not possible, and some materials such as metal oxides and carbon 30 having DFNS morphology cannot be synthesized using this method so some experimental conditions such as co-surfactant and reaction conditions should be modified.^47^

### 2.2. Microemulsions

Microemulsions contain isotropic liquid mixtures of water, oil, surfactant, and co-surfactant, which were first reported by Hoar *et al.* In microemulsions, the surfactant molecules align themselves to form spherical aggregates in the continuous phase. This method, including water-in-oil (w/o) microemulsions, or reverse micelles, was used for the preparation of ultrafine silica NPs. The silica NPs were developed inside the water droplets of a w/o microencapsulation, catalyzed by the base-assisted hydrolysis of silicon alkoxide. The major disadvantage reported in this approach is the high cost and difficulty in the removal of surfactants in the final products. Jin-Lee *et al.* showed that in the first stage, cetyl pyridinium bromide and urea were dissolved in water, then cyclohexane and iso-propanol were added to the solution. Next, TEOS was added dropwise to the mixed solution. After that, the obtained solution was heated to 70 °C for 16 h. In the final stage, the obtained NPs were washed with ethanol until the surfactants were removed from the surface of the NPs.^32^

### 2.3. Sol–gel method

The sol–gel method is a wet-chemical technique that is widely explored for the synthesis of silica NPs with uniform size.^40–42,47^ In the coating process, it involves the hydrolysis and condensation of TEOS or inorganic salts such as sodium silicate (Na_2_SiO_3_) in the presence of a mineral acid or base as the catalyst, or under alkaline conditions in ethanol solution.^43^ Zhang *et al.* illustrated the preparation DFNS by the sol–gel method. In the first stage, tetraethyl orthosilicate (TEOS) and cetyltrimethylammonium tosylate (CTA^+^) were dissolved in water. Then, the reaction was carried out at 80 °C for 2 h, then the obtained NPs were filtered and dried.^48^

### 2.4. Optimized conditions for the synthesis of DFNS

Several studies have been done for optimizing the synthesis protocol of DFNS. Table 2 compares the different conditions used for the preparation of DFNS with various sizes and textural properties by changing some reaction parameters. It has been found that precise control over the size, shape, and surface properties could be achieved by varying the reaction parameters, such as pH, temperature, and surfactant chemical nature, and controlling the condensation of the silica source. Table 2 also shows the type of surfactant and solvent used in the chemical reaction; the co-surfactant and reaction conditions are important parameters that can impact the size of the DFNS and inter-wrinkle distances by controlling them to process different sizes of DFNS. The particle size and inter-wrinkle distances are two main characterization parameters of DFNS for obtaining suitable types in biomedical studies. For example, Moon and Lee succeeded in synthesizing DFNS with smaller size (50–500 nm) by using isopropanol or n-butanol as the co-surfactant and increasing the time of reaction in the microemulsion model.^32,49^ Another parameter illustrated the use of organic amine (instead of urea) and cetyltrimethylammonium (CTA^+^) tosylate and bromide as templates to make DFNS with size <200 nm.^48^ Following this study, small-sized DFNS (<50 nm) was also synthesized by using nonionic surfactants like 1-butyl-3-methylimidazolium trifluoromethane sulfonate ([BMIM] OTF), or pluronic F127, which can stop the growth size of silica particles.^50^ Experimental data showed that using a cationic base such as lysine instead of urea and ethanol as co-surfactants made very small DFNS with size of 14 nm.^51^ On the other hand, for making large-sized DFNS, it is better to use cationic surfactants like CPB and non-ionic co-surfactants like urea.^52^ This study showed that increasing the temperature and reaction time could produce DFNS with small size of approximately 14 nm.^53^

**Table 2:** A comparison of the different conditions used for the preparation of DFSN

Surfactant	Si-Precursor	Solvent	Cosurfactant	Base	Particle size (nm)	Reaction condition	Ref.
CTAB or CPB	TEOS^e^	Water : cyclohexane	1-Pentanol	Urea	90–120	120 °C, 4 h	37 and 122
CPB	TEOS	Water : cyclohexane	Isopropanol or *n*-butanol	Urea	50–500	70 °C, 16–20 h	32 and 49
CTAB^a^	TEOS	Water : toluene	*n*-Butanol	Urea	50–500	120 °C, 4 h	136
CTAT^b^	TEOS	Water	None	Organic amines	<200	80 °C, 2 h	48
CTAT	TEOS	Water	[BMIM] OTF^g^	Triethanolamine	40–100	80 °C, 3 h	50
CPB^c^	TEOS	Water : cyclohexane	Urea	Urea	200–500	120 °C, 20 h	52
CTAC^d^	TEOS	Water : 1-octadecene	Triethanolamine	Triethanolamine	180	60 °C, 12 h	137
CTAB	TEOS	Water : ethylether	Ethanol	Aqueous ammonia	100–320	20 °C, 4 h	138
CTAB	TEOS	Water : octane and styrene	Ethanol	Lysine, AIBA^f^	40–50	70 °C, 20 h	51
CPB	TEOS	Water/cyclohexane	1-Pentanol	Urea	14.78	120 °C, 6 h	53

a Cetyltrimethyl ammonium bromide.

b Cetyltrimethyl ammonium tosylate.

c Cetylpyridinium bromide.

d Cetyltrimethylammonium chloride.

e Tetra ethyl ortho silicate.

f 2,2′-Azobis(2-methylpropionamidine) dihydrochloride.

g 1-Butyl-3-methylimidazolium trifluoro-methanesulfonate.

## 3. Characterization techniques

Different techniques have been the main methods for the characterization of DNFSs. For instance, to verify the synthesis of silica NP-based mesoporous materials, we can use Fourier transform infrared spectroscopy (FTIR),^54^ X-ray diffraction (XRD) analysis,^55^ transmission electron microscopy (TEM),^56^ scanning electron microscopy (SEM),^37^ atomic force microscopy (AFM), Brunauer–Emmett–Teller (BET) analysis,^57^ dynamic light scattering (DLS),^37^ thermogravimetric analysis (TGA),^50^ X-ray photoelectron spectroscopy (XPS),^58^ energy-dispersive X-ray fluorescence spectroscopy (ED-XRF),^53^ vibrating sample magnetometer (VSM),^59,60^ solid-state 13C CPMAS,^61^ and 29Si CPMAS NMR spectroscopy.^53,62^ The functional groups of nanocomposites can be identified by FTIR, and XRD (used for the determination of the crystalline structure and the phase purity of modified silica NPs). SEM, DLS, TEM, and AFM techniques are used to check the size, size distribution and morphology of the NPS. By utilizing more sophisticated microscopy techniques, surface images can be constructed *via* a physical probe that scans the specimen in order to view the chemical composition near the surface of the nanocomposites. TGA is used for measuring the thermal stability of the monomers and coating the surface of the nanomaterial, the moisture content and the possible decomposition of volatile materials in the nanostructured catalysts. XPS is a surface-sensitive quantitative spectroscopy technique that measures the elemental composition in the range of parts per thousand, and also helps to determine the oxidation state and chemical connectivity of the metal present in the nanomaterial. The metal present in the heterogeneous catalyst can be detected *via* ED-XRF, and the magnetic proration of NPs is assessed by VSM. Solid-state NMR spectroscopy is used to verify the covalent grafting of organic functionalities onto silica NP surfaces, and BET analysis is used to investigate the surface, pore size, and pore volume of DFNS.^63^

## 4. Functionalization of DFNS for tumor targeting purposes

The surface of DFNS is a main factor that directly affects the interaction of the DFNS with tumor cells. DFNS has been applied in the biomedical and pharmaceutical fields in target therapy and drug delivery. The DFNS have special shapes that allow them to easily interact with damaged cells. For instance, the DFNS surfaces have large amounts of (Si–OH) groups that allow covalent conjugation with various types of functional groups, including carboxylates, amines, amines/phosphonates, polyethylene glycol, octadecyl, and carboxylate/octadecyl groups.^64^ The solubility of the drug in biological environments is also an important factor in drug delivery. The shape of DFNS allows them to release highly water-soluble anticancer drugs in aqueous biological media. On the other hand, when the drugs have hydrophobic properties, the DFNS provide control over the drug release in aqueous surroundings, releasing the drug in contact with hydrophobic media. The functional groups on the surface of DFNS can be made to specifically target the DFNS with targeting ligands to mediate the internalization of DFNS into the cells. The greatest advantage of DFNS, other than being mesoporous nanoparticles, is the high loading capacity of the drug, which makes them suitable for tumor therapy. The blood circulation time and the ability of the DFNS to overcome biological barriers make them effective for targeting. Some properties such as size, shape, surface charge, and the composition of the DFNS will affect the penetration, tissue accumulation and cellular uptake. The studied articles illustrated that the best size for DFNS to enter tissues is less than 400 nm; DFNS with a larger size than 400 nm cannot diffuse into the tumor in sufficient quantities to get the therapeutic effect.^45^

## 5. Biosafety study of DFNS

The toxicity of nanomaterials is a major concern in terms of *in vivo* and clinical applications. Developed NSs should possess preferred efficacy with no/trivial undesired biological functions. While there is no common agreement on the safety of silica-based materials, the toxicity of these types of nanomaterials should be deeply investigated prior to their use in the clinic. Indeed, the size, surface properties and morphology of MSNs are the main important contributing factors that may have significant effects on the biocompatibility and biosafety of DFNS. Some cytotoxicity assessments have shown that the smaller the size of the DFNS, the greater the toxicity would be. Altogether, the smaller DFNS at higher concentrations may induce greater cell damage and elicit profound hemolytic effects, in large part due to the enhanced uptake as compared to the larger DFNS. The *in vitro* evaluation of DFNS toxicity showed that the larger DFNS can easily escape from the endo/lysosomal compartments. Despite these findings, a shared vision regarding the biosafety of DFNS should be reached among scientists in terms of their size effects. The pore size of DFNS may also have direct impacts on their toxicity and hemolytic activity. Accordingly, various studies have been conducted, showing that the smaller the pore size, the greater the hemolytic activity. This phenomenon may happen due to the fewer interactions of the silanol groups of DFNS with greater pore size with RBCs, as compared to nonporous particles. Of note, DFNS-based DDSs show slow biodegradability of inorganic materials, which might limit the applications of MSNs-based systems, particularly in terms of the matrix degradation-related release of loaded anticancer agents. It has been shown that by inserting functional organosilica groups into the framework of mesoporous silica, the degradation of the mesoporous silica can be accelerated by external stimuli. In a study directed by Shao *et al.*, dual-responsive and biodegradable diselenide-bridged DFNS were fabricated for protein delivery in cancer therapy. They capitalized on the diselenide-bond-containing organosilica precursor for the synthesis of organo-bridged DFNS loaded with ribonuclease A (RNase A). The surface of RNase A-loaded DFNS was subsequently coated with cancer cell membranes (CM) to supply cancer targeting and immune-evasion ability. The results showed that the NS can release its cargo through a matrix degradation mechanism in response to the oxidative/redox conditions in TME.

The surface properties of DFNS are another factor that can directly affect their biosafety. Although the positive charge of the DFNS surface causes an enhanced uptake, it can definitely increase the cytotoxicity and biological damage as compared to anionic/neutral particles. Some modifications such as PEGylation of DFNS may prevent their aggregation and opsonization and hence, decrease their immune clearance and cytotoxicity while prolonging their systemic circulation period. The accelerated blood clearance (ABC), which may be activated after repeated administration of PEGylated particles, needs to be carefully controlled. The lipid coating modification of DFNS is another strategy for reducing the toxicity, which can also improve the pharmacokinetic (PK) properties of NPs attributable to the reduced quantity of non-specific protein bindings (plasma protein opsonization) and prolonged systemic circulation time.

The morphology of DFNS can also directly influence their biosafety. Studies have shown that the endocytosis of DFNS with spherical shape is relatively higher than the cylindrical MSNs with the same surface area. However, some other studies have shown that the level of endocytosis is highly dependent on the type of cells. The aspect ratio is another determinant for the biosafety of DFNS, in which the greater aspect ratio might lead to enhanced uptake and improved biological activity. The higher surface area is a suitable physical property for the encapsulation of cargo in MSNs, even though it may have adverse effects on the safety of DFNS through the increase in the generation of reactive oxygen species (ROS) as DFNS facilitates ROS generation *via* their exposed surface areas. Thus, the higher surface area may result in a higher toxicity of DFNS. In this context, spheres and short rod DFNS can cause greater mitochondrial damage, resulting in an intensified generation of intracellular ROS. Collectively, while various observations confirmed the pivotal role of the size, shape, surface morphologies, and structure on the biosafety of MSNs, more investigations need to be undertaken to address the precise effects of these factors on the biosafety of DFNS.

The safety of the nanoparticles has led to the need for the field of therapy to be characterized according to *in vivo* protocols based on the nanomaterials' absorption, distribution, metabolism, excretion, and toxicity properties; however, few studies have discussed their *in vivo* biodistribution and toxicity after oral administration. Yu *et al.* found that the appearance of DFNS, such as their porosity and surface characteristics, may affect the acute toxicity in immune-competent mice and found that there was *in vivo* toxicity after injection. Also, by increasing the dose of MSNs' (aspect ratio 1, 2, 8) at approximately 30–65 mg kg^−1^, there was mechanical clogging of the vasculature followed by organ failure. These findings indicate that the MSNs' with larger hydrodynamic size have the maximum tolerated dose (MTD).^65^ In another study done by Soleymani *et al.*, the cytotoxicity of Tb@KCC-1-NH_2_-FA on colorectal cancer cells was evaluated by MTT assay, which confirmed their biocompatible nature^66^ This analysis showed that DFNS had no cytotoxic effects on colon cells. The next aspect to consider about DFNS is that their toxicity properties depend on the surface functionalization and amount injected into the body. To date, DFNS has not been evaluated in clinical trials; they have only been tested in different animal models.^67^ All of the articles considered in this review confirmed the safety of DFNS in animal models.

## 6. Biomedical application of DFNS

### 6.1. Cell therapy

Cell therapy involves the injection, grafting, or implanting of viable cells into a patient in order to achieve a therapeutic effect.^68^ Designing intelligent NPs with high biocompatibility and cell uptake is necessary for cell therapy.^69^ In the new studies, DFNS synthesis in water/oil biphasic systems can be used as a platform for biomedical applications. Head–tail mesoporous silica nanoparticles (HTFSNs) or DFNS with controllable structures have been synthesized in oil/water emulsion systems. Also, spherical silica head particles with two features, namely a solid or porous nature, can be used to increase the size of a dendritic tail, as compared to other nanomaterials. HTFSNs illustrated surprisingly higher hemocompatibility toward red blood cells because they covered large dendritic pores. HTFSNs were further tested for immuno–adjuvant activity. Interestingly, Yu and co-workers found that asymmetrical HTFSNs demonstrated a higher rate of uptake and *in vitro* maturation of antigen-presenting cells as compared to spherical head particles. This study has provided a new idea for the use of nanocarriers in vaccine development and immunotherapy; the change in RBC structure was studied by SEM and TEM apparatus.^70^

### 6.2. Gene therapy

Gene therapy is a promising therapeutic strategy to combat many serious gene-related diseases such as cancer, monogenic disorders, cardiovascular diseases, Parkinson's disease, cystic fibrosis and muscular degeneration.^40,71^ Gene therapy is used for the treatment of cancers by enhancing the expression of tumor suppressor genes in a desired time frame.^72^ It has high efficiency and common usage in biomedical applications. Nevertheless, it may have some essential drawbacks including immunological problems, insertional mutagenesis and limitations in the size of the carried therapeutic genes; therefore, a carrier that can deliver the correct dose of the gene to the recipient has always been the focus of attention. In recent years, polymeric nanolipids and peptide carriers have been extensively studied.^71,73–75^ The use of DFNS surface-modified with sensitive pH polymers is very important for the release of genes on the target cell. Qiao *et al.* showed that when AC-PEI (polyethyleneimine@acetaldehyde-cystine) was functionalized with DFNS, the RNA could be easily released into the cells.^76^ In another study, the capacity of several nanoparticles including MCM-41 and KCC-1 for the adsorption of DNA was investigated. First, MCM-1 and KCC-1 were synthesized and then their surfaces were functionalized with amine groups. The prepared nanocomposites included derivative compounds from KCC-1 and MCM-41, such as KCC–NH_2_, KCC–NH–NH_2_, KCC–CPB and MCM–41, MCM–NH_2_, MCM-41–CTAB. Then prepared nanocomposites were studied for the adsorption of DNA. The results showed that the derivative compounds from the fibrous, large pore KCC-1 had a high capacity for the adsorption, transport, and delivery of DNA and genes as compared to MCM-41.^40^

### 6.3. Immune therapy

#### 6.3.1. Cancer immune therapy

DFNS has extensive potential as a delivery vehicle for biomolecules due to the morphology, uniform fibrous structure, high surface areas, and biocompatibility. In a new study by Yual *et al.*, poly(I:C) was combined with stellated fibrous MS nanospheres and then the synergistic effects were investigated for decreasing the necessary dose of poly(I:C) for cancer immunotherapy. The results showed an average particle size of about 100–200 nm in diameter and open pores up to 35 nm. Moreover, the combination of poly(I:C) on the surface of DFNS showed high adsorption affinity for biomolecules, high delivery ability for transporting biomolecules into BMDCs and immune-stimulating effects in the *in vitro* experiment. The nanocomposite was tested on mice that were immunized with MS–OVA-PIC, which showed a more significant increase in anti-cancer immunity as compared to those immunized with free OVA-PIC at a similar dose. Also, the mice immunized with MS–OVA-PIC (poly(I:C) dose of 0.625 mg kg^−1^) showed comparable anti-cancer immunity to those immunized with 4 times the dose (2.5 mg kg^−1^) of free poly(I:C). All of the data, illustrated as MS–OVA-PIC, opened up great opportunities due to the future clinical applications of poly(I:C) in cancer immunotherapy.^70^

In another study, Hanagata *et al.* synthesized a new DFNS that can be used as carrier cytosine-phosphate-guanosine oligodeoxynucleotide (CpG ODNs).

One of the nucleic acids with powerful immuno-stimulatory activity is CpG ODNs, with numerous medical applications such as the treatment of cancer, allergies, asthma, and infectious diseases.^77,78^ However, the poor stability and cellular uptake efficiency of free CpG ODNs lead to poor immune stimulatory activity. In this study, DFNS was synthesized with average particle size between 97–500 nm, then the surface was modified with 3-APTS (3-aminopropyltriethoxysilane). Next, Raw 264.7 cells were cultured with the DFNS–NH_2_–based CpG ODN delivery system, and the *in vitro* cytotoxicity, cellular uptake, and TLR-9 mediated induction of IL-6 were studied. The obtained results indicated that the DFNS–NH_2_–based CpG ODN had the highest IL-6 induction ability (96–1 ± 1–6 μg mg^−1^) as compared to free CpG ODN.^79^

Changing the conformation of DFNS could give new insight for deeper studies on the applications of DFNS. New generations of DFNS like the double-shelled dendritic mesoporous organosilica hollow spheres (DMSOHS-*n*S) were synthesized by Yu *et al.*^80^ (DMOHS-1S) and (DMOHS-2S) were synthesized and simultaneously used in tumor cell studies. This study indicates that (DMOHS-2S) could open new doors for developing advanced vaccine delivery systems for cancer therapy.^81^

### 6.4. Drug delivery

#### 6.4.1. Anticancer drug delivery

Nanotechnology can be introduced as a technology that develops materials or devices within the nanometer scale. This technology can be very helpful in targeted drug delivery and therapeutics for disease prevention, diagnosis, and treatment.^82^ Nanocarriers such as liposomes, micelles, polymeric nanocarriers, dendrimers, hydrogels, metallic nanocarriers, QDs, carbon-based nanocarriers, exosomes, and viruses have been extensively studied for the design of drug delivery systems.^80,83–93^ The design of nanocarriers for drug delivery offers several advantages, including the improvement of hydrophobic drug stability, thereby making them plausible for administration, the enhancement of biodistribution, and pharmacokinetics. This results in improved efficacy, improvement of the EPR effect, increased selective targeting, reduced adverse effects as a consequence of favored accumulation at target sites, and decreased toxicity by using biocompatible nanomaterials.^82^ KCC-1 is of great interest in drug delivery; due to the fibrous morphology of DFNS, the use of nanocarriers in drug delivery could be facilitated. The silica layer of DFNS could be used as a platform for drug releases in various conditions. As a result, DFNS has become a viable alternative for the loading and mass transfer efficiency, even for larger biomolecules such as proteins, enzymes, DNA, siRNA, *etc.* On the other hand, nanosilica spheres have a significant disadvantage of the limited surface area for the loading of biomolecules.

In 2012, Gai *et al.* succeeded in delivering DOX in *in vitro* conditions by DFNS. Firstly 0.5 μg ml ^−1^ of DOX was prepared in PBS solution (pH = 7.4) under dark conditions. Then, 0.6 mg of DFNS was added to the solution and shaken for 24 h. The obtained DFNS nanoparticles were separated by centrifugation then dissolved in water and determined by UV spectroscopy at 490 nm; the results showed that the average recovery of the nanoadsorbent was 90%.^94^ In another study, Du *et al.* synthesized dendrimer-like amino-functionalized silica nanoparticles with hierarchical pores (HPSNs-NH2) and the surface was modified by COOH, PEI, and NH_2_ groups.^95^ Following this study, Munaweera *et al.* managed to make periodic mesoporous organosilica (PMO) nanoparticles for the delivery of the hydrophobic anticancer drug (Paclitaxel) in PBS solution (pH = 7.4).^20^

The treatment of cancers using different types of DFNS armed with targeting moieties (*e.g.*, small molecules, peptides, proteins, antibodies, or Ap) seems to be one of the most promising cancer therapy modalities that might be practically translated into clinical applications in the near future. These NSs may offer much lower off-target side effects with maximal therapeutic impacts on the target cells/tissues. Aps, synthetic peptides, or short-length single-strand oligonucleotide molecules recognize and bind to their specific targets with high specificity. Therefore, Aps might be the favored candidate ligand in the targeted therapy of cancer. They are, however, associated with some limitations (*e.g.*, vulnerability to the nuclease enzymes), which make their usefulness questionable. The conjugation of Aps on the surface of nanomaterials is facilitated by its unique chemistry, which may lead to a successful targeted drug delivery against cancer. Nanomaterials and their physicochemical features are the other key crucial components that influence enhanced drug delivery. DFNS possess a wide range of requirements for successful drug delivery due to their advanced physicochemical features such as the well-organized porous structure, the higher volume of pores and surface area, and low *in vivo* toxicity. Recently, several investigations have been directed toward developing various smart Ap-decorated DFNS with the robust abilities of enhanced cancer therapy. Providentially, some efforts have shown promising capabilities in various types of DDSs. Considering the exclusive physicochemical and PK/PD features of Ap-decorated DNFS, they are expected to be practically utilized for targeted therapy with decreased off-target side effects. Despite all these, this field is still growing and many crucial issues remain to be addressed prior to the translation of Ap-conjugated DFNS into clinical applications.

#### 6.4.2. Protein drug delivery using DFNS

**Fig. 2:**
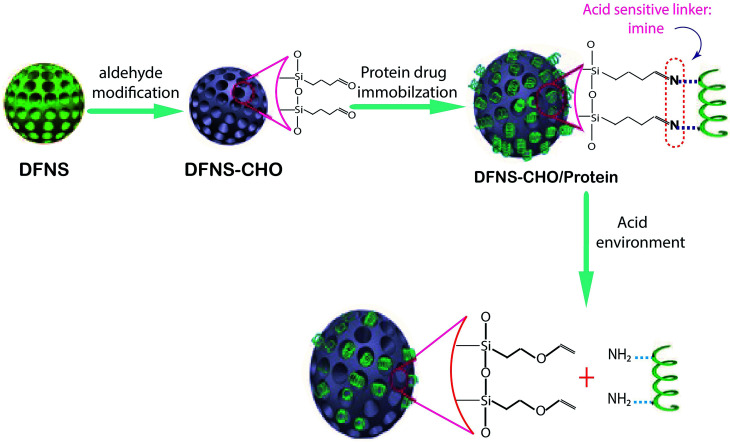
Schematic representation of the construction of the pH-responsive protein drug delivery system based on DFNS nanoparticles.^96^

DFNS have been great biocarriers due to their easy synthesis, surface functionalization, special structure, biocompatibility and chemical stability. The unique structures of DFNS including open dendritic superstructures with large mesopore channels have led to DNA delivery, RNA delivery and some biological drugs such as IFN-α. In a new study, DFNS with an average particle size of 174 ± 17 NM was prepared, then the surface was functionalized with aldehyde groups. Fig. 2 shows the preparation of (DFNS–CHO). The release of bovine serum albumin (BSA) on the surface of (DFNS–CHO) was investigated at different pHs. Furthermore, the *in vitro* cytotoxicity and cell uptake of the DFNS-CHO nanoparticles were studied using RAW264.7 cells as the cellular system. The results indicated that the nanocarriers had a highly loaded capacity of approximately 136 μg mg^−1^. Moreover, in acidic conditions, BSA was released from the DFNS–CHO nanoparticles with no cytotoxicity. It was concluded that DFNS–CHO nanoparticles would be acceptable as novel nanocarriers for the pH-responsive delivery of protein drugs.^96^

One of the main injected drugs for type 1 and type 2 diabetes is insulin; its use is sometimes quite painful for patients. Finding an oral type of insulin is important in pharmaceutical science. Kleitz *et al.* found a new oral drug delivery route for insulin *via* DFNS. In the first stage, DFNS with positive charges in various pore sizes have been designed and negatively charged insulin was loaded on the surface of DFNS. The surfaces of the nanoparticles were then modified with pH-sensitive protein (B-lactoglobulin). The data showed that with this method, insulin was release at pH 7.4.^97^

#### 6.4.3 Ayurvedic drug delivery using DFNS

**Fig. 3:**
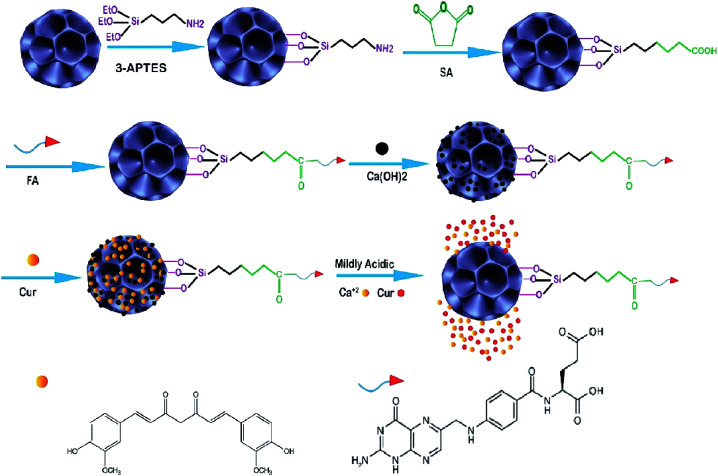
Schematic illustration of the fabrication of Cur–Ca@DFNS–FA and the pH-stimuli-responsive release in tumors.^100^

The ayurvedic system of medicine is one of the oldest systems of traditional medicine. Many people prefer to use ayurvedic drugs instead of chemical drugs due to chemical drugs having many side effects. It is always time-consuming to find a new method for the delivery of ayurvedic drugs.^98^ Nanoparticles such as DFNS can encapsulate enormous biomaterials and drugs in their structural pores and deliver them to the target. In this research project, DFNS-based multifunctional nanoparticles were reported as a drug delivery system. Firstly DFNS was prepared by a previously reported method with slight modification;^99^ the average size was 63 nm, and one of the optimal cancer-targeting ligands with a strong chemical attraction to folate receptors (*K*_d_ = 10^−10^ M) is folic acid. In this project, folic acid (FA) was conjugated to the surface of DFNS for selectively targeting cancer cells, then biocompatible calcium hydroxide and curcumin (Cur) were immobilized on (DFNS–FA) (Fig. 3). Finally, the anti-cancer efficacy of obtaining the nanocomposite (Cur–Ca@DFNS–FA) was investigated by a battery of *in vitro* and *in vivo* experiments. The results show that the Cur–Ca@DFNS–FA was diffuse in aqueous solution. Cur was control-released by pH and efficiently targeted the MCF-7 cells of breast cancer. Cur–Ca@DFNS–FA effectively inhibited cell proliferation, enhanced intracellular ROS generation, reduced mitochondrial membrane potential, and increased cell cycle retardation at the G2/M phase, leading to greater apoptosis in MCF-7 as compared to free Cur. On the other hand, the western blotting analysis illustrated that Cur–Ca@DFNS–FA was more effective than free Cur through the suppression of PI3K/AKT/mTOR and Wnt/β-catenin signaling, and the activation of the mitochondria-mediated apoptosis pathway. *In vivo* studies indicated that Ca@DFNS–FA exhibited good biocompatibility, the Cur concentration in blood serum and tumor tissues increased after 1 h when Cur was encapsulated in Ca@DFNS–FA and finally, all of the data showed that Cur–Ca@DFNS–FA was the best candidate against breast cancer.^100^

**Fig. 4:**
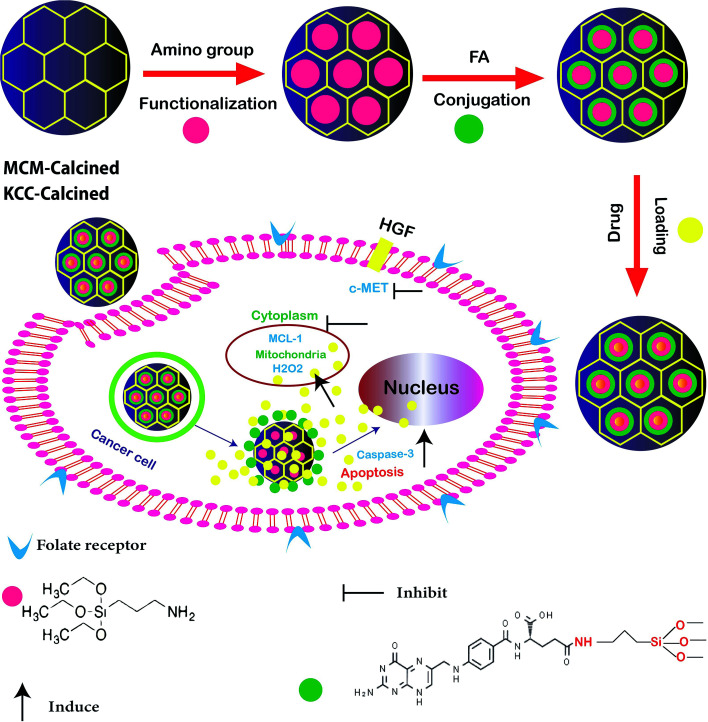
Schematic presentation of the prepared nanosystem from preparation, internalization, and anticancer mechanism of action DFNS in human liver carcinoma (HepG2) cells.^101^

In other research, the scientists focused on the anticancer effect of folic acid conjunction on both surfaces of mesopore nanoparticles, including KCC-1 and MCM. Fig. 4 shows the anticancer mechanism of DFNS in human liver carcinoma (HepG2) in this study; various factors, such as the surface of nanoparticles, timing, and size, which may influence the cellular uptake of NPs by cancer cells were investigated. The kind of surface modification (as-synthesized MSNs, amino-modified MSNs, and FA-conjugated MSNs), and incubation time (4, 12, 24, and 48 h) were investigated on two types of NPS including KCC-1 and MCM. All of the results show that the FA-conjugated KCC-1 has promise for controlling the release of curcumin, quercetin, and colchicine as compared to MCM.^101^

Following this work in 2016, Lojkowski synthesized a new type of mesoporous silica nanoparticles for the controlled release of curcumin (Cur) as an anticancer natural pro-drug in an *in vitro* experiment. Firstly, two types of nanocomposite including KCC–NH_2_ and MCM–NH_2_ were synthesized, then cur was loaded on the surface, and the release of Cur was investigated at several pH values (2.5, 5–7.5) under *in vitro* conditions. The results showed that the highest amount of Cur was released under acidic conditions (pH 2.5). This opens the way for their application in controlling the curcumin delivery in cancer due to the acidic tumor environment increasing its stability and leading to an increase in the Cur bioavailability. The KCC–NH_2_ seems to be a more promising nanocarrier for the release of Cur as compared to the commonly used MCM-41 material.^102^

#### 6.4.4 Antimicrobial enzyme delivery using DFNS

The misuse of antibiotics has led to the appearance of antibiotic-resistant bacteria and dangerous threats to human health.^103^ Lysozyme is an abundant enzyme in nature and has antimicrobial activity towards Gram-positive bacteria by separating the glycoside linkage in the bacterial cell wall. It is less effective towards Gram-negative bacteria and can be limited by its poor stability and weak binding affinity to the surface of bacteria; therefore, it requires a method that can increase its surface area. Yu *et al.* synthesized three types of DFNS with various sizes, including MSNs-FC2-R1, MSNs-FC2-R0.4, MSNs-FC8-R0.15. The results showed that MSNs-FC8-R0.15 had a smaller size and density as compared to other DFNP. The lysozyme-loaded MSNs-FC8-R0.15 carried on complete inhibition toward *E. coli* for five days. The bacterial killing activity was also confirmed by an agar plate test. The fibrous morphology of the nanocarriers improved the bacterial membrane adhesion.^99^

#### 6.4.5 Immobilization of Candida rugosa lipase by DFNS

By developing nanotechnology, nanocomposites such as nanoparticles, nanotubes, and nanofibrous silica particles were used as enzyme immobilization due to their special structures including their high surface area, high stability, and easy separation.^104–106^ Zhang *et al.* synthesized two types of nanocomposite, namely, Fe_3_O_4_@SiO_2_@hollow@fibrous SiO_2_ (yolk shell-1), and Fe_3_O_4_@SiO_2_@hollow@ mesoporousSiO_2_ (yolk shell-2), due to the immobilization of *Candida rugosa* lipase (CRL) through template-assisted selective etching. In the first stage, Fe_3_O_4_ nanoparticles were synthesized. Then, the SiO_2_ shell was coated on the surface of Fe_3_O_4_ by a sol–gel reaction, and the prepared Fe_3_O_4_@SiO_2_ composites were covered by the resorcinol formaldehyde (RF) shell supplied with mesoporous fibrous (KCC-1) in the yolk shell-1. Yolk shell-2 was prepared from yolk shell-1 but the structure of mesoporous silica was not fibrous and glutaraldehyde was used as a linker for the immobilization of *Candida rugosa* lipase. The results indicated that yolk shell-1, with large magnetization (25–29.5 emu g^−1^), highly accessible mesoporous channels (20–50 nm), high surface area (600–400 m^2^ g^−1^), and good loading and enzyme activity as compared to yolk shell-2, was the best candidate for use due to the immobilization of *Candida rugosa*.^107^

### 6.5. Imaging

#### 6.5.1. Bioimaging by DFNS

Quantum Dots (QD) have several forms, one of which is cadmium telluride (CdTe), and they are used in various ways due to their unparalleled size-sensitive optoelectronic properties. However, their photostability and colloidal stability issues, as well as their toxicity, are somewhat limited. Zhang *et al.* used DFNS to stabilize CdTe and enhance its stability and cytotoxicity. 3-APTS was loaded on DFNS to manufacture mSiO_2_–NH_2_. In the next stage, CdTe was functionalized with mSiO_2_–NH_2_ by using the electrostatic attraction between the positively charged surface and the negatively charged CdTe. A modified DFNP was formed over it using Stöber's method to improve its stability. SEM analysis illustrated the dendritic fibrous nature of mSiO_2_@CdTe@SiO_2_, and EDS elemental mapping showed an equal distribution of CdTe on the fibers of DFNS silica. These materials showed enhanced QD stability, low toxicity and strong fluorescence (used for bioimaging).^108^

#### 6.5.2. Real-time imaging using DFNS

Innovative mesoporous nanomaterials have provided new opportunities for cell imaging. They are interesting because of their sensitivity, modularity, capacity for many potentially varied ligands, and their potential for multifunctional imaging.^109^ In a recent study, a novel DFNS was synthesized by a mild one-pot hydrothermal process. In addition to functionalization, the magnetism and fluorescence were amalgamated into a single mesoporous silica composite particle composed of 0.1 g of fluorescein dye and 0.05 g of Fe_3_O_4_ nanoparticles in DFNS. The obtained nanocomposite was able to load drugs and other large molecules. The synthesized nanocomposite was incubated with murine fibroblast L-929 cell lines to examine the cytotoxicity and ability for *in vitro* fluorescence labeling of living cells. These multifunctional nanocomposites with huge specific surface areas were accepted as ideal platforms due to targeted mass drug delivery and tracking based on their magnetic and luminescence properties.^110^ The cytotoxicity study illustrated that these materials can be safely used for live-cell imaging and MRI at concentrations less than 10 ppm. The fluorescence imaging showed that these NPs could be used for cell imaging, where green fluorescence was observed in the cytoplasm as well as inside the nuclei due to their internalization and equal distribution within the cells.

#### 6.5.3. Photoacoustic imaging by DFNS

In recent years, phototheranostic imaging has received significant attention in modern nanomedicine dealing with cancer tumor imaging and therapy. Improving the signals from cancer cells is an important stage for diagnosis. Among the variety of nanocarriers systems, DFNS possesses good size, fluidity, deformability, and great encapsulation ability to integrate different ingredients; this allows DFNS to have a range of theranostic applications.^111^ Every cancer cell has a special genetic code made from microRNA.^112^ MicroRNA plays a significant regulatory role in an array of biological processes and is used as a biomarker for potential diagnosis and forecasting in clinical applications. Dong *et al.* illustrated that when MicroRNA are encapsulated in DFNS and functionalized with DNA photoacoustic (DNA-PA) probes and glutathione (GSH), signal amplification occurs to show the place of cancer cell (MCF-7 cell). It was shown that microRNA, by improving the signal of photoacoustic instruments, have enormous potential in advanced molecular imaging *in vivo*.^113^

### 6.6. Radiotherapy

#### 6.6.1. Delivery of chemo- and radiotherapeutics simultaneously using DFNS

**Fig. 5:**
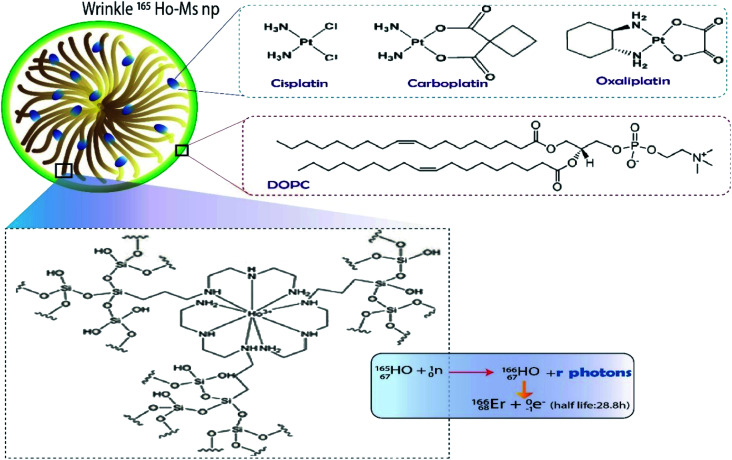
DOPC lipid-coated platinum drug-loaded holmium-165 containing DFNS.^21^

Over the last decade, DFNS was used in the development of a smart delivery vehicle that simultaneously delivered chemo- and radiotherapeutics.^21^ Studies have shown that the loading of radiodrugs on the surface of DFNS could reduce the frequency of use of the radiodrug.^21,114^ Munaweera *et al.* succeeded in synthesizing DFNS that could load 2-dioleoyl-*sn*-glycero-3-phosphocholine (DOPC) lipid-coated/uncoated platinum drug and holmium complex (165Ho-MS np), which becomes radioactive after neutron-activation (166Ho-MS np). Fig. 5 shows the process of loading. The synthesis of nanomaterials illustrated the radioactivity of 213.6 μCi mg^−1^ after neutron activation for 1 h (thermal neutron flux of 3.5 × 1012 neutrons per cm^2^ s) Moreover, platinum drug loading of 14.6, 11.7, and 16.1% (w/w) was achieved for cisplatin. The *in vitro* results showed that the controlled release of platinum drugs from the DFNS was over 40 h for both lipid-coated and uncoated systems. These results indicated that both ^165^Ho-MS-Pt and DOPC–^165^Ho-MS-Pt are promising candidates for combined chemo and radiotherapy.^21^

### 6.7. Photothermal therapy

#### 6.7.1. Photothermal ablation (PTA) therapy using DFNS

**Fig. 6:**
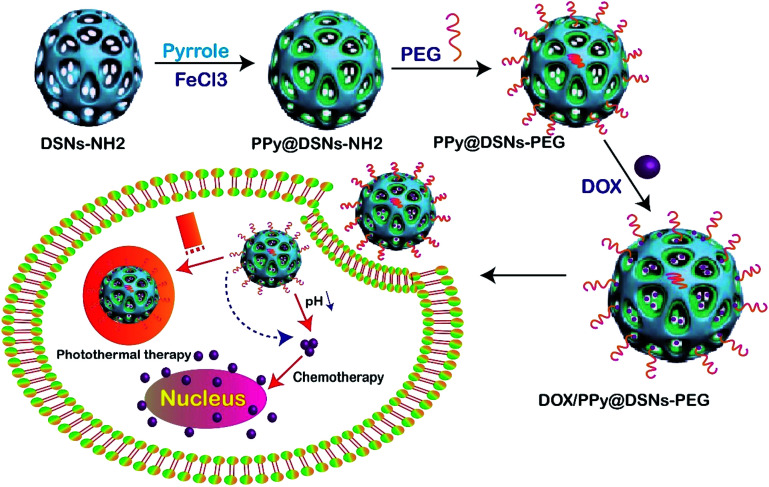
Schematic illustration of the preparation of DOX/PPy@DSNs–PEG for combined chemo-photothermal therapy.^116^

Cancer is a significant public health problem across the world. In recent years, various methods have been used to treat cancer. One of these methods recently used is photothermal ablation (PTA) therapy, which can effectively kill cancer cells in a specific region by converting light energy into heat.^115^ This method has received significant interest as a potentially effective treatment for tumor necrosis as compared to other methods. What makes it an excellent alternative for cancer therapy is that it is non-invasive and targeted. Currently, a large variety of nanocomposite has been explored for photothermal agents based on their high near-infrared (NIR). In this study, PPy@DSNs–NH_2_ was fabricated by functionalized polypyrrole on DFNS, and then modified by PEG to enhance the biocompatibility and stability in physiological conditions. The spatial structure of the mesopore channels of DFNS led to the loading of the anticancer drug (DOX) on the surface fabricated PPy@DSNs–PEG nanocomposite (Fig. 6). Different techniques such as (FESEM), (TEM), (FTIR), (TGA), (DLS), (BET), UV-visible spectroscopy were used to characterize the nanocomposite. Photothermal performance and *in vitro* drug release were also studied, and the synergistic therapeutic efficacy of DOX/PPy@DSNs–PEG was evaluated by *in vitro* MTT assays and confocal microscope observation. The obtained results illustrated that the new nanocomposite DOX/PPy@DSNs–PEG had synergistic chemo-photothermal therapy, physiological stability, and high NIR absorbance, as well as powerful heat transformation. The prepared nanocomposite showed a high DOX loading.^116^

#### 6.7.2 Photodynamic therapy using DFNS

Photodynamic therapy (PDT) is a main-stage treatment that amalgamates light energy with a drug (photosensitizer) to eradicate cancerous and precancerous cells after light activation. Photosensitizers are agitated by a particular wavelength of light energy, mostly by laser. Before being activated by light, the photosensitizer is not toxic but after light activation, the photosensitizer has a toxic effect on the targeted tissue.^117^ Common organic photosensitizers (PSs) are still widely used in cancer treatment in PDT but they have some drawbacks including hydrophobicity, poor stability within the PDT environment and low cell/tissue specificity, which limit their clinical applications. In recent years, the combination of nanoparticles with the PDT method could reduce their side effects. One of the mesoporous nanoparticles introduced by Chengzhong Yu *et al.* for photodynamic therapy was the core–shell-structured dendritic mesoporous silica nanoparticles. In this study, C60 was dispersed in the silica core of DFNS due to photosensitizers and fluorescence agents for imaging. Then, C18 loading to DFNS was used to load a therapeutic mAb anti-pAkt. All of the modifications led to the generation of O_2_ and the fluorescence light excitation in MCF-7 cancer cells. The results indicated the cancer cell inhibition by reducing the levels of anti-apoptotic proteins required by using modified DFNS in combination with the PDT method.^118^

#### 6.7.3 Mediated combination therapy with DFNS

Cancer therapy based on nanomaterials plays an important role in significantly increasing the therapeutic efficiency against cancer by utilizing a combination of chemotherapeutic agents with nanomaterials. Recent findings illustrated that the combination of two or more nanoparticle-mediated therapies can result in a synergistic therapeutic result to improve the current cancer treatments. Combination therapy has also been illustrated to increase tumor control and reduce undesired side effects of cancer drugs by improving the pharmacokinetics and targeted delivery of the drug payloads. The special structure of silica mesoporous nanoparticles allows them to chosen as the best candidates in mediated combination therapy. Yan *et al.* succeeded at synthesizing hollow dendritic mesoporous silica nanoparticles capped with chitosan (GM-CS) then functionalized with folic acid (HDFSNs-GM-CS-FA). They used these NPs for the co-delivery of pheophorbide (PA) and doxorubicin (DOX). HDFSNs-GM-CS-FA@DOX/PA fully combined photothermal, photodynamic and chemotherapies to develop synergistic antitumor efficacy.^119^

In another study, DOX was loaded into DFSN then coated with AgNPs. The synthesized DOX/AgNPs/DFSN was injected to cancerous cells *via* endocytosis. In the next stage, DFNS was released into cytosol by endosomal pH through endosomal-lysosomal escape. The released AgNPs destroyed the mitochondrial tissue, then DOX penetrated the DNA, which resulted in DNA damage by the poisoning of topoisomerase II. Both AgNPs and DOX induced cancer cell death through oxidative stress by DNA damage in the nucleus.^91^

### 6.8. Bioanalysis

#### 6.8.1. Biosensors

**Fig. 7:**
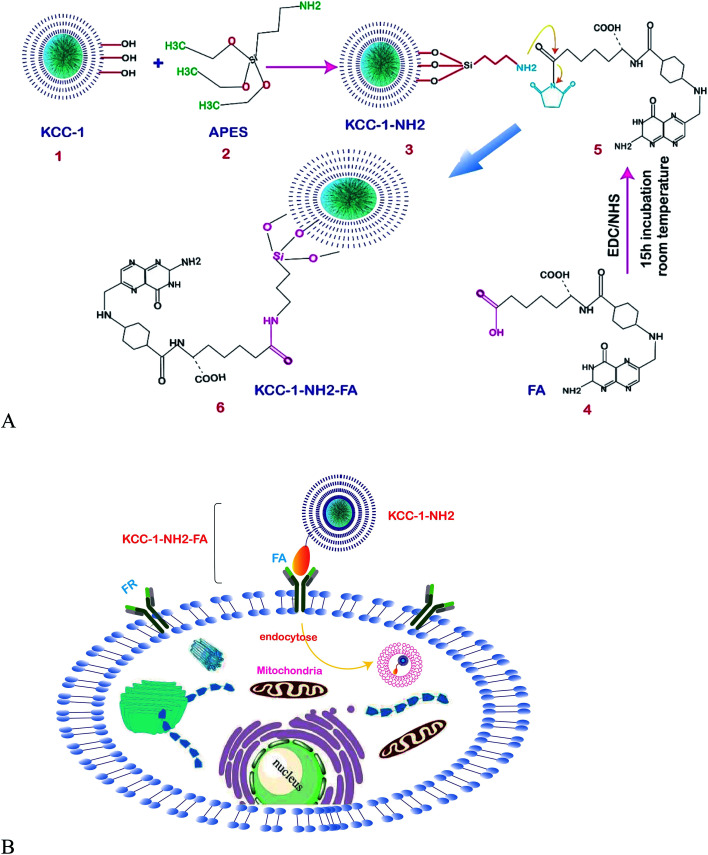
(A) Schematic representation of the functionalization of KCC-1-NH_2_. (B) Schematic illustration of the mechanism of the developed FR-positive cytosensor.^121^

The highly sensitive sensing of cancer cells in a short time is very important in clinical studies. One of the main stages in biosensor analysis is the fabrication of the surface of the electrode. The unique physicochemical properties of these fibrous nanomaterials make them excellent candidates in electrochemical and optical biosensing. One of the biomarkers investigated in colon carcinoma is HT29.^120^ In a new study, Soleymani *et al.* successfully determined HT29 in FR-positive center cells by folic acid functionalized with KCC-1-NH_2_ nanoparticles. Fig. 7A shows the stages for functionalization and Fig. 7B shows the mechanism of the developed FR-positive cytosensor. Two sensitive electrochemistry techniques SWV and DPV were used for the detection. In addition, flow cytometry and fluorescence imaging were used to determine the quantitative and qualitative attachment of KCC-1-NH_2_-FA to the HT 29 FR-positive cancer cells. The obtained data showed that the cytosensor response was in a linear range from 50 to 12 000 cells per ml with LLOQ of 50 cells/ml. Therefore, the cytosensor is accurate and simple, providing perfect specificity and sensitivity, which will enable us to design point of care devices for clinical use.^121^

One of the amino acids that were classified as a non-essential amino acid is l-proline (l-Pro); the biological and biotechnological applications are clear for any biomedical research program, and the introduction of sensitive and easy methods for its determination in biological samples is in demand. Mirzaie and colleagues succeeded at making an innovative electrochemical biosensor for the sensitive and specific detection of l-proline (l-Pro) in human plasma for the first time. KCC-1 was synthesized by the method by Bayal *et al.*,^122^ then functionalized with3-APTES to prepare KCC-1-NH_2_ as the enzyme encapsulating support. The fabrication of the surface of the electrode was done by encapsulating PRODH/POX on KCC-1-NH_2_, and l-proline was finally determined by the ultra-sensitive electrochemistry methods of cyclic voltammetry and chronoamperometry in PBS at pH = 7. The results showed that the fabricated electrode can determine l-Pro in the range of 2.26 to 15 mM, with a LLOQ of 2.26 mM. Therefore, due to the proper function and high sensitivity of the prepared enzyme biosensor, this method can be used to encapsulate and increase the temperature stability of the enzyme as well as to detect l-Pro.^123^

**Fig. 8:**
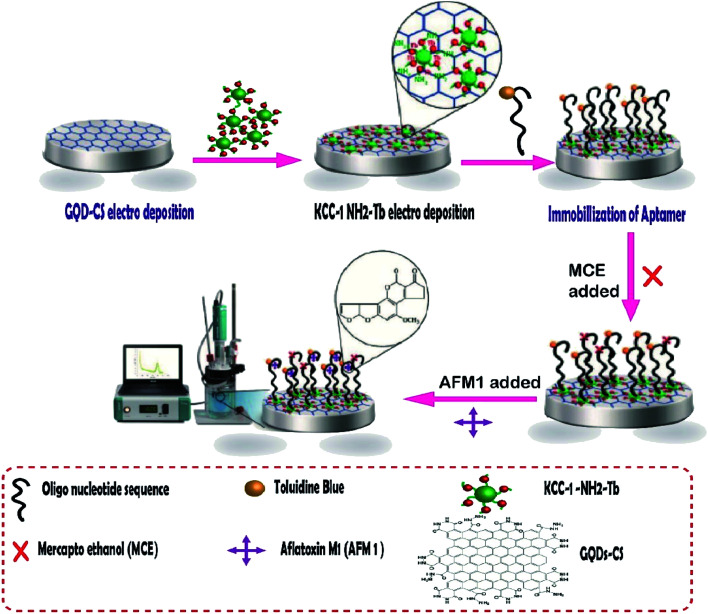
The aptasensor assembly procedure and its application in the monitoring of AF M1.^125^

Aflatoxins are among the food pollutant items produced by fungi, the most toxic of which is aflatoxin M1 (AFM1). It is a human carcinogen that is found in milk products and may have potentially severe health impacts on milk consumers. In addition, the cancer risk has been reportedly caused by AFM1 in small amounts.^124^ Aflatoxin M1 was determined by an aptamer-based biosensor whose surface was modified by dendritic fibrous nanosilica functionalized by amine groups. In this project, the research group attempted to design a novel, creative electrochemical biosensor for the rapid and sensitive quantification of AFM1 in milk samples. To fabricate this biosensor, GQD-CS and KCC-1–NH_2_–Tb were synthesized, and then the nanocomposites were electrodeposited on the surface of GCE. Next, the special oligonucleotide sequence, as a probe aptamer tag with toluidine blue as an indicator, was immobilized on the surface of GQDs-CS/KCC-1–NH_2_–Tb (Fig. 8). Two ultra-sensitive techniques, namely, cyclic voltammetry (CV) and differential pulse voltammetry (DPV), were used for the determination of AFM1 in real samples. The results showed that the method is in the linear range of 0.1 μM to 1 fM with LLOQ of 10 fM.^125^

#### 6.8.2. Bio-analysis

Untreated water can cause many gastrointestinal illnesses. To have a safe, clean environment, the removal of the toxic organic compounds in water is vital. In recent years, many nanoadsorbents have been introduced to solve this problem. In a new study by Polshettiwar *et al.*, diamino-functionalized KCC-1 (DA-KCC-1) with micro-mesostructured fibers was chosen as a nanoadsorbent for the removal of Congo red from water samples. Essential parameters were investigated and optimized, such as the initial pH value, adsorbent dose, and initial dye concentration. The maximum uptake capacity was found to be ∼400 mg g^−1^, according to the Langmuir model.^126^

**Fig. 9:**
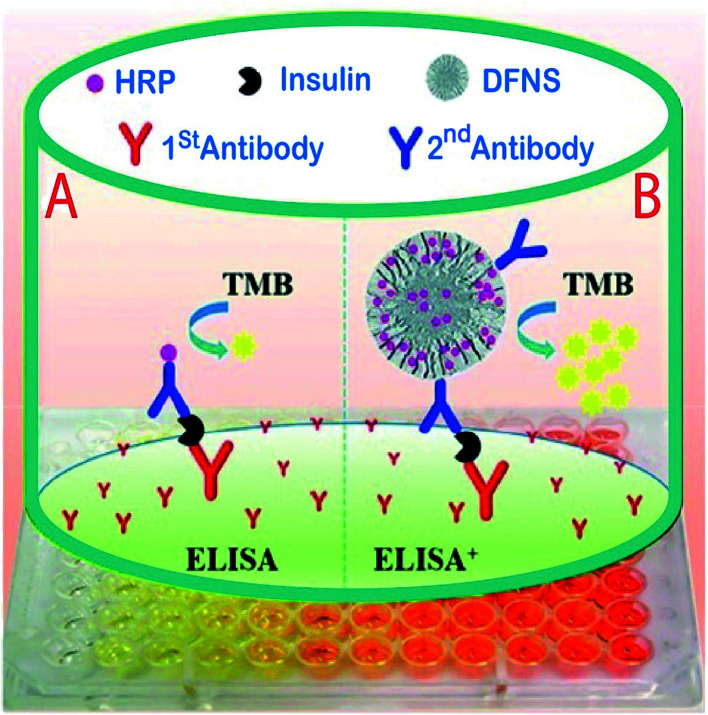
A comparison of (A) traditional sandwich-like ELISA and (B) the ELISA+ using modified DFNS with high potential HRP loading and achievable dendritic channels to obtain ultrasensitive detection.^127^

In another study for the determination of insulin in human serum samples, an ultrasensitive enzyme-linked immunosorbent assay (ELISA^+^) was fabricated by using dendritic fibrous silica nanoparticles (DFSN) with high horseradish peroxidase (HRP) loading and easily accessible pore channels for signal amplification. In this project, Yu *et al.* successfully developed three different pore diameters, 6.9 nm (DFNS-1), 14.9 nm (DFNS-2), and 34.2 nm (DFNS-3) by immobilizing the horseradish peroxidase (HRP) enzyme by treating it with aldehyde functionalized DFNS *via* Schiff base linkage (Fig. 9). The results showed DFNS as having a surface area of 484 m^2^ g^−1^ and a pore volume of 1.39 cm^3^ g^−1^, with an average pore size of 14.5 nm. There were huge loadings of the HRP enzyme, approximately 2000, and it was more sensitive than a commercial ELISA kit for insulin detection in serum. Also, the calibration parameter illustrated that insulin samples could be determined in low concentrations (20–100 fg ml^−1^) with LOQ of 7.7 pg ml^−1^ and a recovery rate of 81% was achieved;^127^ compared to the other reported analytical methods,^128,129^ the new method has an acceptable recovery and LOQ.

#### 6.8.3. Bioseparation by DFNS

Protein phosphorylation, a spacious protein post-translational modification, has been implicated in numerous important cellular activities such as molecular recognition, signaling transduction and metabolic processes.^130–132^ The investigation of protein phosphorylation under various physiological conditions is an essential stage in understanding the signaling pathways. The mechanism of disease generation and diagnosis, and the detection of phosphorylated proteins in bio-samples are the important stages. In a new study, after the synthesis of DFNS modified by polydopamine and chelating Ti^4+^, the structure of the obtained nanocomposite was investigated. The results showed a high surface area of 362 m^2^ g^−1^, a large pore volume of 1.37 cm^3^ g^−1^, and a large amount of chelated Ti^4+^ (75 μg.mg^−1^). Compared to other conventional mesoporous silica-based nanomaterials, the LC/MS results illustrated that DFNS@PDA–Ti^4+^ had high selectivity, a lower detection limit (0.2 fmol μl^−1^) and a high specificity (>95%). In addition, the reported nanocomposite identified 2422 unique phosphor peptides from HeLa cell extracts.^133^

### 6.9. Biocatalysis

#### 6.9.1. Application of DFNS as artificial enzymes

**Fig. 10:**
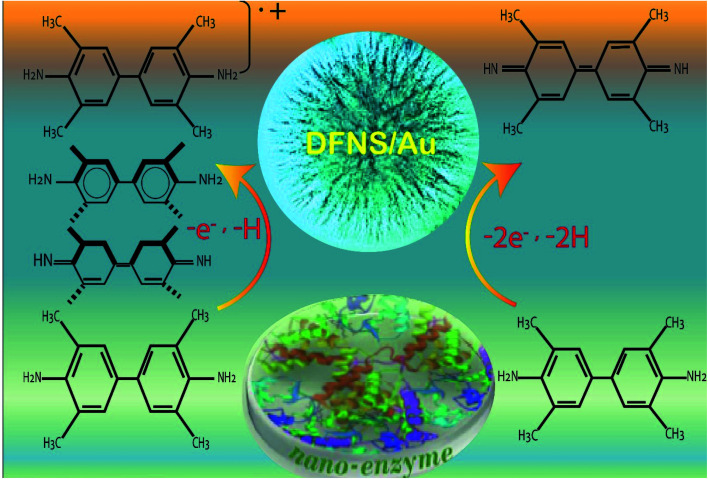
Application of DFNS/Au as an artificial enzyme.^134^

New biocatalysts with 3D structures, which are highly sensitive and stable are in high demand. The design and preparation of natural enzymes are quite challenging for different reasons such as being time-consuming, the high cost of preparation, purification and storage, and difficulties in recovery and recycling. As such, new methods are greatly needed for the preparation of artificial enzymes that have catalytic activities and greater stability. Polshettiwar and his research group succeeded in synthesizing dendritic fibrous nanosilica (DFNS)-supported gold (Au) nanoparticles (DFNS/Au) as artificial enzymes comparable to peroxidases. It was shown that the new artificial enzyme had high enzymatic activity due to 3,5,3′,5′-tetramethylbenzidine (TMB) oxidation as a model reaction, which was considerably better than the natural horseradish peroxidase (HRP) enzyme. This study shows that DFNS/Au had higher enzymatic activity than other nanocomposites due to the fibrous morphology and unique silica channels. (Fig. 10) shows the stages of the oxidation of TMB by DFNS/Au.^134^

### 6.10. Tissue-engineering

The use of mesoporous silica nanoparticles in tissue engineering is a newly emerged field that has attracted much research interest^135^ but studies were not accomplished for the investigation DFNS in tissue engineering until now.^136–139^

## 7. Conclusion

This review has mainly focused on the biomedical applications of dendritic fibrous silica nanoparticles. We briefly discussed the synthesis methods and various conditions used for the preparation of DFNS, and toxicity issues related to their medical use. We have compared the physicochemical properties of different mesoporous nanoparticles such as MCM-41, SBA-15, Stöber silica, DFNS, and MSN, and discussed the characterization methods used for investigating the structure of DFNS. The articles studied show that DFNS has unique structures with large numbers of active sites with extraordinarily high accessibility as compared to conventional mesoporous silica materials. Due to the radially oriented pores (channels), the size of DFNS can be manipulated. Biomaterials can quickly access the active sites on the channels of DFNS and increase their interactions with the functionalized organic substances on the surface of DFNS. The biomedical application of DFNS was investigated in drug delivery, therapy, bioanalysis, biocatalysis, bioimaging and biotechnologies. The obtained results showed that for real clinical applications, these nanoprobes must have excellent biocompatibility, good colloidal stability, long circulation time, and controllable size range.

## 8. Future perspectives

DFNS are an emerging class of mesoporous silica nanoparticles (MSNPs) and are used as biomaterial in various biomedical fields. Due to their unique structure, they can interact with a range of polymers, and macromolecules. They have great biocompatibility and cytocompatibility. Numerous biomedical applications of DFNS have been scientifically explored, such as cell therapy, gene therapy, immunotherapy, drug delivery, chemotherapy, photothermal therapy, imaging, bioanalysis, and biocatalysis. Future studies will focus on the development of these techniques toward clinical applications. Many of these studies are currently not sufficient and just involve the testing of lab animals. On the other hand, the interactions of DFNS with cells are not significant and several parameters such as pore size, channel size, pH and toxicity need to be investigated. We expect that omics-based approaches, including proteomics, transcriptomics and metabolomics will be investigated for the interactions of DFNS with cells. Many MSNPs are used to increase drug solubility but there are no similar studies on the application of DFNS in the solubility of drugs. Therefore, there is the opportunity for the investigation of drug solubility studies based on DFNS. Also, in chemotherapy, it has been illustrated that DFNS can reduce the amount of radiodrug used for therapy; *i.e.*, the side effects of radio drugs will be reduced by these methods. Computational modeling software can be used to estimate the interactions between DFNS with cells or biopolymers. By understanding these interactions, custom inks for 3D printing can be produced. Another exciting application of DFNS is in tissue engineering. It would be interesting to investigate the use DFNS for bone tissue, vascular tissue and wound healing. DFNS can interact with bone cells, skin cells and others, but it is not clear why and how DFNS have these properties. It would be valuable to investigate this phenomenon for further potential applications in tissue engineering.

Finally, novel DFNS has three-dimensional dendritic structures with large pore channels and highly accessible internal surface areas for various applications. We expect to increase the cellular uptake and improve the interactions by changing the shape, size and charge of DFNS. All of the parameters facilitate the different biomedical applications of the nanoparticles in the near future.

## Conflicts of interest

There is no conflict of interest.

## Supplementary Material

## References

[cit1] Yao C. G., Martins P. N. (2020). Transplantation.

[cit2] Lee D. W., Yoo B. R. (2016). J. Ind. Eng. Chem..

[cit3] Wang Y., Zhao Q., Han N., Bai L., Li J., Liu J., Che E., Hu L., Zhang Q., Jiang T. (2015). Nanomedicine.

[cit4] Hasanzadeh M., Shadjou N., de la Guardia M., Eskandani M., Sheikhzadeh P. (2012). TrAC, Trends Anal. Chem..

[cit5] Agrahari V. (2017). Neural Regener. Res..

[cit6] Solhi E., Hasanzadeh M. (2019). TrAC, Trends Anal. Chem..

[cit7] Vasudevan S. M., Ashwanikumar N., Kumar G. V. (2019). Biomater. Sci..

[cit8] Hasanzadeh M., Shadjou N., Eskandani M., de la Guardia M. (2012). TrAC, Trends Anal. Chem..

[cit9] Wang Y., Du X., Liu Z., Shi S., Lv H. (2019). J. Mater. Chem. A.

[cit10] Maity A., Polshettiwar V. (2017). ChemSusChem.

[cit11] Mahmudi M., Shadjou N., Hasanzadeh F. A. M. (2019). J. Electroanal. Chem..

[cit12] Tzankova V., Aluani D., Yordanov Y., Valoti M., Frosini M., Spassova I., Kovacheva D., Tzankov B. (2019). Drug Chem. Toxicol..

[cit13] Downing M. A. and Jain P. K., in Nanoparticles for Biomedical Applications, Elsevier, 2020, pp. 267–281

[cit14] Zahiri M., Babaei M., Abnous K., Taghdisi S. M., Ramezani M., Alibolandi M. (2019). J. Cell. Physiol..

[cit15] Li T., Shi S., Goel S., Shen X., Xie X., Chen Z., Zhang H., Li S., Qin X., Yang H. (2019). Acta Biomater..

[cit16] Xu C., Lei C., Yu C. (2019). Front. Chem..

[cit17] Nguyen T. L., Choi Y., Kim J. (2019). Adv. Mater..

[cit18] Xie Y., Wang J., Wang M., Ge X. (2015). J. Hazard. Mater..

[cit19] Califano V., Sannino F., Costantini A., Avossa J., Cimino S., Aronne A. (2018). J. Phys. Chem. C.

[cit20] Munaweera I., Hong J., D'Souza A., Balkus K. J. (2015). J. Porous Mater..

[cit21] Munaweera I., Koneru B., Shi Y., Di Pasqua A. J., Balkus J., Kenneth J. (2014). APL Mater..

[cit22] Huang X., Townley H. (2016). Nanobiomedicine.

[cit23] Wang Z., Balkus Jr K. J. (2017). Catal. Commun..

[cit24] Munaweera I., Shi Y., Koneru B., Patel A., Dang M. H., Di Pasqua A. J., Balkus Jr K. J. (2015). J. Inorg. Biochem..

[cit25] Qian T., Li J., Min X., Deng Y., Guan W., Ning L. (2016). Energy.

[cit26] Gao J., Kong W., Zhou L., He Y., Ma L., Wang Y., Yin L., Jiang Y. (2017). Chem. Eng. J..

[cit27] Wan D., Yan C., Liu Y., Zhu K., Zhang Q. (2019). Microporous Mesoporous Mater..

[cit28] Zhao F., Wang X., Ding B., Lin J., Hu J., Si Y., Yu J., Sun G. (2011). RSC Adv..

[cit29] Mogharabi-Manzari M., Ghahremani M. H., Sedaghat T., Shayan F., Faramarzi M. A. (2019). Eur. J. Org. Chem..

[cit30] Liu Z., Ru J., Sun S., Teng Z., Dong H., Song P., Yang Y., Guo H. (2019). J. Mater. Chem. B.

[cit31] Du X., He J. (2011). Langmuir.

[cit32] Moon D.-S., Lee J.-K. (2012). Langmuir.

[cit33] Du X., Zhao C., Zhou M., Ma T., Huang H., Jaroniec M., Zhang X., Qiao S. Z. (2017). Small.

[cit34] Mizoshita N., Tani T., Inagaki S. (2011). Chem. Soc. Rev..

[cit35] Park S. S., Moorthy M. S., Ha C.-S. (2014). NPG Asia Mater..

[cit36] Wang Y., Du X., Liu Z., Shi S. (2019). J. Mater. Chem. A.

[cit37] Polshettiwar V., Cha D., Zhang X., Basset J. M. (2010). Angew. Chem., Int. Ed..

[cit38] Zhang S., Qian Y., Ahn W.-S. (2019). Chem. Sci..

[cit39] Maity A., Belgamwar R., Polshettiwar V. (2019). Nat. Protoc..

[cit40] Huang X., Tao Z., Praskavich Jr J. C., Goswami A., Al-Sharab J. F., Minko T., Polshettiwar V., Asefa T. (2014). Langmuir.

[cit41] Shahangi F., Chermahini A. N., Saraji M. (2018). J. Energy Chem..

[cit42] Mohammadbagheri Z., Chermahini A. N. (2018). J. Ind. Eng. Chem..

[cit43] Hasan R., Chong C. C., Setiabudi H. D. (2019). Bull. Chem. React. Eng. Catal..

[cit44] Hasan R., Chong C., Bukhari S., Jusoh R., Setiabudi H. (2019). J. Ind. Eng. Chem..

[cit45] Rahikkala A., Pereira S. A., Figueiredo P., Passos M. L., Araújo A. R., Saraiva M. L. M., Santos H. A. (2018). Adv. Biosyst..

[cit46] Daou T., Pourroy G., Bégin-Colin S., Grenèche J.-M., Ulhaq-Bouillet C., Legaré P., Bernhardt P., Leuvrey C., Rogez G. (2006). Chem. Mater..

[cit47] Maity A., Belgamwar R., Polshettiwar V. (2019). Nat. Protoc..

[cit48] Zhang K., Xu L.-L., Jiang J.-G., Calin N., Lam K.-F., Zhang S.-J., Wu H.-H., Wu G.-D., Albela B. l., Bonneviot L. (2013). J. Am. Chem. Soc..

[cit49] Moon D.-S., Lee J.-K. (2014). Langmuir.

[cit50] Yu Y.-J., Xing J.-L., Pang J.-L., Jiang S.-H., Lam K.-F., Yang T.-Q., Xue Q.-S., Zhang K., Wu P. (2014). ACS Appl. Mater. Interfaces.

[cit51] Gustafsson H., Isaksson S., Altskär A., Holmberg K. (2016). J. Colloid Interface Sci..

[cit52] Yang H., Liao S., Huang C., Du L., Chen P., Huang P., Fu Z., Li Y. (2014). Appl. Surf. Sci..

[cit53] Sadeghzadeh S. M., Zhiani R., Khoobi M., Emrani S. (2018). Microporous Mesoporous Mater..

[cit54] Sadeghzadeh S. M. (2016). Appl. Organomet. Chem..

[cit55] Shahul Hamid M. Y., Triwahyono S., Jalil A. A., Che Jusoh N. W., Izan S. M., Tuan Abdullah T. A. (2018). Inorg. Chem..

[cit56] Qureshi Z. S., Sarawade P. B., Albert M., D'Elia V., Hedhili M. N., Köhler K., Basset J. M. (2015). ChemCatChem.

[cit57] Aghakhani A., Kazemi E., Kazemzad M. (2015). J. Nanopart. Res..

[cit58] Gautam P., Dhiman M., Polshettiwar V., Bhanage B. M. (2016). Green Chem..

[cit59] Yu K., Zhang X., Tong H., Yan X., Liu S. (2013). Mater. Lett..

[cit60] Hassankhani A., Sadeghzadeh S. M., Zhiani R. (2018). RSC Adv..

[cit61] Fihri A., Cha D., Bouhrara M., Almana N., Polshettiwar V. (2012). ChemSusChem.

[cit62] Lilly Thankamony A. S., Lion C., Pourpoint F., Singh B., Perez Linde A. J., Carnevale D., Bodenhausen G., Vezin H., Lafon O., Polshettiwar V. (2015). Angew. Chem., Int. Ed..

[cit63] Sharma R., Sharma S., Dutta S., Zboril R., Gawande M. B. (2015). Green Chem..

[cit64] Pereira S. A. P. (2018). Adv. Biosyst..

[cit65] Yu T., Greish K., McGill L. D., Ray A., Ghandehari H. (2012). ACS Nano.

[cit66] Soleymani J., Hasanzadeh M., Shadjou N., Somi M. H., Jouyban A. (2020). J. Pharm. Biomed. Anal..

[cit67] Mamaeva V., Sahlgren C., Lindén M. (2013). Adv. Drug Delivery Rev..

[cit68] Newick K., O'Brien S., Moon E., Albelda S. M. (2017). Annu. Rev. Med..

[cit69] Andre E. M., Passirani C., Seijo B., Sanchez A., Montero-Menei C. N. (2016). Biomaterials.

[cit70] Abbaraju P. L., Meka A. K., Song H., Yang Y., Jambhrunkar M., Zhang J., Xu C., Yu M., Yu C. (2017). J. Am. Chem. Soc..

[cit71] Loh X. J., Lee T.-C., Dou Q., Deen G. R. (2016). Biomater. Sci..

[cit72] Liu J., Song L., Liu S., Jiang Q., Liu Q., Li N., Wang Z.-G., Ding B. (2018). Nano Lett..

[cit73] Ye H., Owh C., Loh X. J. (2015). RSC Adv..

[cit74] Loh X. J., Zhang Z.-X., Mya K. Y., Wu Y.-l., He C. B., Li J. (2010). J. Mater. Chem..

[cit75] Loh X. J., Ong S. J., Tung Y. T., Choo H. T. (2013). Macromol. Biosci..

[cit76] Du X., Xiong L., Dai S., Kleitz F., Qiao S. Z. (2014). Adv. Funct. Mater..

[cit77] Li J., Pei H., Zhu B., Liang L., Wei M., He Y., Chen N., Li D., Huang Q., Fan C. (2011). ACS Nano.

[cit78] Krieg A. M. (2002). Annu. Rev. Immunol..

[cit79] Xu Y., Zhu Y., Li X., Morita H., Hanagata N. (2016). Mater. Express.

[cit80] Benyettou F., Rezgui R., Ravaux F., Jaber T., Blumer K., Jouiad M., Motte L., Olsen J.-C., Platas-Iglesias C., Magzoub M. (2015). J. Mater. Chem. B.

[cit81] Yang Y., Lu Y., Abbaraju P. L., Zhang J., Zhang M., Xiang G., Yu C. (2017). Angew. Chem., Int. Ed..

[cit82] Nanjwade B. K. , Sarkar A. B. and Srichana T., in Characterization and Biology of Nanomaterials for Drug Delivery, Elsevier, 2019, pp. 337–350

[cit83] Agrawal A. K., Aqil F., Jeyabalan J., Spencer W. A., Beck J., Gachuki B. W., Alhakeem S. S., Oben K., Munagala R., Bondada S. (2017). Nanomedicine.

[cit84] Altunbas A., Lee S. J., Rajasekaran S. A., Schneider J. P., Pochan D. J. (2011). Biomaterials.

[cit85] Duman F. D., Erkisa M., Khodadust R., Ari F., Ulukaya E., Acar H. Y. (2017). Nanomedicine.

[cit86] Galaway F. A., Stockley P. G. (2012). Mol. Pharmaceutics.

[cit87] Hua S., Vaughan B. (2019). Int. J. Nanomed..

[cit88] Smith B. R. and Ghosn E., US Patent App, 16/353743, 2019

[cit89] Thi H., Thanh T., Nguyen Tran D.-H., Bach L. G., Vu-Quang H., Nguyen D. C., Park K. D., Nguyen D. H. (2019). Pharmaceutics.

[cit90] Kuruvilla S. P., Tiruchinapally G., Crouch A. C., ElSayed M. E., Greve J. M. (2017). PLoS One.

[cit91] Watermann A., Brieger J. (2017). Nanomaterials.

[cit92] Batrakova E. V., Haney M., Klyachko N., Zhao Y., Kabanov A. (2018). J. Extracell. Vesicles.

[cit93] Prasad S. R., Kumar T. S., Jayakrishnan A. (2017). Nanotechnology.

[cit94] Gai S., Yang P., Wang L., Li C., Zhang M., Jun L. (2012). Dalton Trans..

[cit95] Du X., Shi B., Liang J., Bi J., Dai S., Qiao S. Z. (2013). Adv. Mater..

[cit96] Tian Z., Xu Y., Zhu Y. (2017). Mater. Sci. Eng. C.

[cit97] Juère E., Caillard R., Marko D., Del Favero G., Kleitz F. (2020). Chem.–Eur. J..

[cit98] Farooq S. , Mehmood Z., Qais F. A., Khan M. S. and Ahmad I., in New Look to Phytomedicine, Elsevier, 2019, pp. 581–596

[cit99] Wang Y., Nor Y. A., Song H., Yang Y., Xu C., Yu M., Yu C. (2016). J. Mater. Chem. B.

[cit100] Wang J., Wang Y., Liu Q., Yang L., Zhu R., Yu C., Wang S. (2016). ACS Appl. Mater. Interfaces.

[cit101] AbouAitah K., Swiderska-Sroda A., Farghali A. A., Wojnarowicz J., Stefanek A., Gierlotka S., Opalinska A., Allayeh A. K., Ciach T., Lojkowski W. (2018). Oncotarget.

[cit102] AbouAitah K. E., Farghali A., Swiderska-Sroda A., Lojkowski W., Razin A., Khedr M. (2016). J. Nanomed. Nanotechnol..

[cit103] Wang Y., Wang Y., Li X., Li J., Su L., Zhang X., Du X. (2018). ACS Sustainable Chem. Eng..

[cit104] Xie W., Wang J. (2014). Energy Fuels.

[cit105] Jamshaid T., Neto E. T. T., Eissa M. M., Zine N., Kunita M. H., El-Salhi A. E., Elaissari A. (2016). TrAC, Trends Anal. Chem..

[cit106] Shaban M., Hasanzadeh M., Solhi E. (2019). Anal. Methods.

[cit107] Ali Z., Tian L., Zhang B., Ali N., Khan M., Zhang Q. (2017). Enzyme Microb. Technol..

[cit108] Zhang S., Wen L., Yang J., Zeng J., Sun Q., Li Z., Zhao D., Dou S. (2016). Part. Part. Syst. Charact..

[cit109] Wang P., Kim T., Harada M., Contag C., Huang X., Smith B. R. (2020). Nanoscale Horiz..

[cit110] Atabaev T. S., Lee J. H., Lee J. J., Han D.-W., Hwang Y.-H., Kim H.-K., Hong N. H. (2013). Nanotechnology.

[cit111] Zhang C. , Li D. and Shi X., in Photonanotechnology for Therapeutics and Imaging, Elsevier, 2020, pp. 23–43

[cit112] Dong H., Dai W., Ju H., Lu H., Wang S., Xu L., Zhou S.-F., Zhang Y., Zhang X. (2015). ACS Appl. Mater. Interfaces.

[cit113] Zhang K., Meng X., Yang Z., Cao Y., Cheng Y., Wang D., Lu H., Shi Z., Dong H., Zhang X. (2019). Adv. Mater..

[cit114] Li X.-D., Wang Z., Wang X.-R., Shao D., Zhang X., Li L., Ge M.-F., Chang Z.-M., Dong W.-F. (2019). Int. J. Nanomed..

[cit115] Wang S., Yin Y., Song W., Zhang Q., Yang Z., Dong Z., Xu Y., Cai S., Wang K., Yang W. (2020). J. Mater. Chem. B.

[cit116] Chen R., Yang F., Xue Y., Wei X., Song L., Liu X. (2016). RSC Adv..

[cit117] Falk-Mahapatra R. and Gollnick S. O., Photochemistry and Photobiology, 2020 10.1111/php.13253 PMC7293553 32128821

[cit118] Abbaraju P. L., Yang Y., Yu M., Fu J., Xu C., Yu C. (2017). Chem.–Asian J..

[cit119] Yan T., He J., Liu R., Liu Z., Cheng J. (2020). Carbohydr. Polym..

[cit120] Li H., Su J., Jiang J., Li Y., Gan Z., Ding Y., Li Y., Liu J., Wang S., Ke Y. (2019). Int. J. Biol. Macromol..

[cit121] Soleymani J., Hasanzadeh M., Somi M. H., Shadjou N., Jouyban A. (2019). Biosens. Bioelectron..

[cit122] Bayal N., Singh B., Singh R., Polshettiwar V. (2016). Sci. Rep..

[cit123] Mirzaie A., Saadati A., Hassanpour S., Hasanzadeh M., Siahi-Shadbad M., Jouyban A. (2019). Anal. Methods.

[cit124] Ahlberg S., Grace D., Kiarie G., Kirino Y., Lindahl J. (2018). Toxins.

[cit125] Moosavy M.-H., Hasanzadeh M., Soleymani J., Mokhtarzadeh A. (2019). Anal. Methods.

[cit126] Soltani R., Marjani A., Moguei M. R. S., Rostami B., Shirazian S. (2019). J. Mol. Liq..

[cit127] Lei C., Xu C., Nouwens A., Yu C. (2016). J. Mater. Chem. B.

[cit128] Liu J.-M., Yan X.-P. (2011). J. Anal. At. Spectrom..

[cit129] Hess C., Thomas A., Thevis M., Stratmann B., Quester W., Tschoepe D., Madea B., Musshoff F. (2012). Anal. Bioanal. Chem..

[cit130] Zhao M., Deng C., Zhang X. (2014). Chem. Commun..

[cit131] Yan Y., Zhang X., Deng C. (2014). ACS Appl. Mater. Interfaces.

[cit132] Zhong H., Xiao X., Zheng S., Zhang W., Ding M., Jiang H., Huang L., Kang J. (2013). Nat. Commun..

[cit133] Hong Y., Yao Y., Zhao H., Sheng Q., Ye M., Yu C., Lan M. (2018). Anal. Chem..

[cit134] Singh R., Belgamwar R., Dhiman M., Polshettiwar V. (2018). J. Mater. Chem. B.

[cit135] Chen L., Zhou X., He C. (2019). Wiley Interdiscip. Rev.: Nanomed. Nanobiotechnol..

[cit136] Febriyanti E., Suendo V., Mukti R., Prasetyo A., Arifin A., Akbar M., Triwahyono S., Marsih I. (2016). Langmuir.

[cit137] Shen D., Yang J., Li X., Zhou L., Zhang R., Li W., Chen L., Wang R., Zhang F., Zhao D. (2014). Nano Lett..

[cit138] Du X., Li X., Huang H., He J., Zhang X. (2015). Nanoscale.

[cit139] Maity A., Polshettiwar V. (2018). ACS Appl. Nano Mater..

